# Impact of Transglutaminase-Mediated Crosslinking on the Conformational Changes in a Dual-Protein System and IgE Reactivity of Soy Protein

**DOI:** 10.3390/molecules29143371

**Published:** 2024-07-18

**Authors:** Guangliang Xing, Tianran Hui, Jia Liu, Siran Yang

**Affiliations:** 1 School of Biology and Food Engineering, Changshu Institute of Technology, Changshu 215500, China; 2 UCL Division of Medicine, University College London, London WC1E 6BT, UK; 3 Department of Biological and Environmental Sciences, Troy University, Troy, AL 36082, USA

**Keywords:** transglutaminase, soy protein, sodium caseinate, conformational changes, IgE reactivity

## Abstract

Transglutaminase (TGase)-catalyzed crosslinking has gained substantial traction as a novel strategy for reducing allergenic risk in food proteins, particularly within the realm of hypoallergenic food production. This study explored the impact of TGase crosslinking on conformational changes in a binary protein system composed of soy protein isolate (SPI) and sodium caseinate (SC) at varying mass ratios (10:0, 7:3, 5:5, 3:7 (*w*/*w*)). Specifically, the immunoglobulin E (IgE) binding capacity of soy proteins within this system was examined. Prolonged TGase crosslinking (ranging from 0 h to 15 h) resulted in a gradual reduction in IgE reactivity across all SPI-SC ratios, with the order of IgE-binding capability as follows: SPI > SPI5-SC5 > SPI7-SC3 > SPI3-SC7. These alterations in protein conformation following TGase crosslinking, as demonstrated by variable intrinsic fluorescence, altered surface hydrophobicity, increased ultraviolet absorption and reduced free sulfhydryl content, were identified as the underlying causes. Additionally, ionic bonds were found to play a significant role in maintaining the structure of the dual-protein system after crosslinking, with hydrophobic forces and hydrogen bonds serving as supplementary forces. Generally, the dual-protein system may exhibit enhanced efficacy in reducing the allergenicity of soy protein.

## 1. Introduction

The growing recognition of proteins’ nutritional and functional significance has sparked a surge in interest in recent years, particularly in the development of innovative and nutritionally enhanced food products. This trend involves the integration of diverse protein sources of both plant and animal origin, such as whey protein isolate (WPI), casein, and soy protein isolate (SPI) [1,2]. However, these common ingredients are also known to be primary food allergens, posing risks to susceptible individuals who may experience reactions ranging from mild skin irritation to life-threatening anaphylaxis [3]. To mitigate this concern, the development of dual-protein food products that minimize allergenicity is essential.

Allergies to soy and cow milk often manifest as immunoglobulin E (IgE)-mediated type I hypersensitivity reactions [4]. Researchers have pinpointed 33 soy proteins responsible for IgE binding, with molecular weights varying from 7.5 kDa to 97 kDa [5], with β-conglycinin (7S), glycinin (11S), Gly m Bd 28K (P28), and Gly m Bd 30K (P34) being key allergenic components. In milk, β-lactoglobulin, α-lactalbumin, and casein are the most prevalent and allergenic proteins [6]. Understanding and disrupting allergenic epitopes in soy and milk proteins is crucial for mitigating their allergenic potential.

A multitude of strategies have been developed to address the allergenicity of soy and milk proteins, including chemical modifications, fermentation, enzymatic treatments, heat processing, biotechnological approaches, high-pressure processing, and genetic engineering [7,8]. Among these, enzyme crosslinking, particularly using transglutaminase (TGase; EC 2.3.2.13), has gained significant attention for reducing the allergenicity of soy and milk protein allergens [9,10]. TGase-mediated crosslinking is highlighted as a non-thermal enzymatic approach that effectively reduces allergenicity by promoting the formation of macromolecular aggregates, which can potentially inhibit mast cell activation and alleviate allergic reactions [11]. This method is preferable over genetic engineering, which may require extensive regulatory approval, and over chemical modifications, which may leave harmful residues. Additionally, TGase crosslinking maintains the nutritional and functional properties of proteins, making it more suitable for food applications [12].

While numerous studies have investigated the allergenicity of individual soy and milk proteins, there is limited research on dual-protein systems and their interactions. The objective of this research is to investigate the impact of TGase crosslinking on the structure and IgE reactivity of a dual-protein system comprising SPI and sodium caseinate (SC) in varying ratios. SPI is chosen for its high protein content, with known allergenic components, and prevalence in food products, making it a significant allergen of interest. SC, on the other hand, is a milk-derived protein that is highly soluble and functional in various food systems. The combination of these proteins in a dual-protein system aims to leverage their beneficial properties while investigating their interactions and the potential reduction in allergenicity through TGase crosslinking. Measurements of free sulfhydryl content, sodium dodecyl sulfate-polyacrylamide gel electrophoresis (SDS-PAGE), determination of intermolecular forces, intrinsic fluorescence analysis, ultraviolet absorption spectrophotometry, and surface hydrophobicity assessments were conducted. Additionally, the effect of TGase crosslinking on the IgE reactivity of soy proteins within the dual-protein system was examined using an enzyme-linked immunosorbent assay (ELISA). This investigation is distinct in its focus on crosslinking as a method to structurally modify allergenic proteins and its application within a mixed-protein matrix, a relatively unexplored area in allergen research. It provides insights into reducing the allergenicity of soy protein in a dual-protein system and seeks to enhance the understanding of the interactions between protein components from different sources.

## 2. Results and Discussion

### 2.1. SDS-PAGE

**Figure 1:**
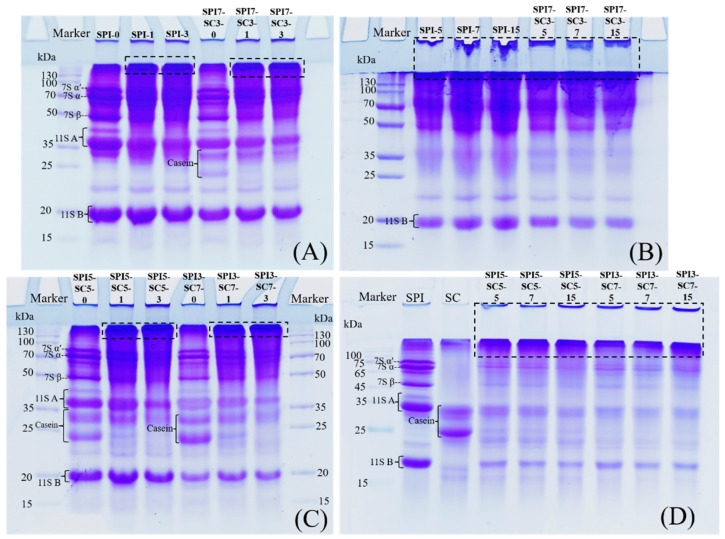
Electrophoretic patterns of dual-protein systems with different SPI-SC ratios incubated at 50 °C for various time durations with the addition of 5 U/g TGase. 7S α’, 7S α and 7S β: subunits of β-conglycinin; 11S A and 11S B: acidic and basic subunits of glycinin. SPI, SPI7-SC3, SPI5-SC5 and SPI3-SC7 refer to the mass ratios of SPI and SC in the dual-protein samples as 10:0, 7:3, 5:5 and 3:7, respectively. SPI-0, SPI-1, SPI-3, SPI-5, SPI-7, SPI-15 refer to SPI samples that cross-linked by TGase for 0, 1, 3, 5, 7, 15 h, respectively, the same for numbering the other dual-protein samples.

The electrophoretic profiles of the dual-protein samples with varying SPI-SC ratios, after incubation at 50 °C in the presence of TGase, were analyzed (Figure 1). The major soy protein, β-conglycinin (7S), consists of subunits α′, α, and β, while glycinin (11S) is categorized into acidic (11S A) and basic subunits (11S B) [13]. Casein comprises three distinct subunits, namely, α-casein, β-casein, and κ-casein [14]. These protein subunits were well separated in the electrophoretogram. As the crosslinking time increased, there was a progressive decrease in the densities of 7S, 11S A, and casein in the samples. Notably, high-molecular-weight protein aggregates (>130 kDa) formed above the stacking gel in all samples (identified by black dashed lines in Figure 1), signifying the occurrence of TGase-mediated crosslinking reactions. It can be observed from Figure 1A,C that the majority of soy protein and casein subunits were mostly crosslinked within 3 h. In Figure 1B,D, it can be noted that with an extended crosslinking time of 15 h, the 11S B subunits in all sample groups remained distinctly visible, suggesting their relative resistance to TGase crosslinking compared to 7S, 11S A, and casein subunits. This finding is consistent with that of previous research [15]. Studies have indicated that under the action of TGase, the 11S basic subunits are less prone to crosslinking compared to the 11S acidic subunits [16,17]. This may be related to the quaternary structure of 11S globulin and the low glutamine/lysine content in the 11S B subunits, which are precisely the active sites of TGase [18,19].

### 2.2. TGase Crosslinking on the Ultraviolet Spectrum of Dual-Protein Samples 

The ultraviolet (UV) absorption characteristics of specific amino acids, such as tryptophan (Trp), tyrosine (Tyr), and phenylalanine (Phe), exhibit absorption peaks in the 220–320 nm wavelength range [20]. These characteristics make it possible to detect alterations in protein structure by examining changes in absorption peaks and absorbance. 

**Figure 2:**
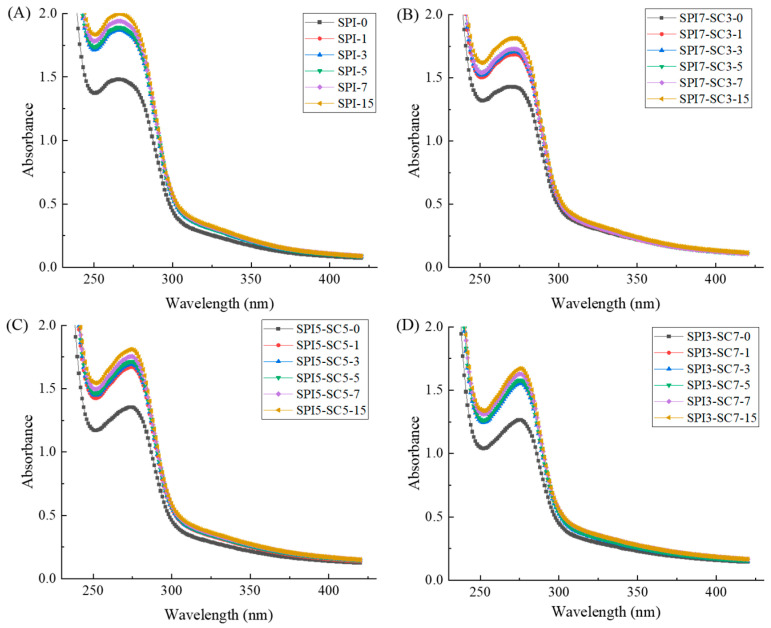
Ultraviolet absorption spectra of dual-protein systems with different SPI-SC proportions ((**A**) SPI, (**B**) SPI7-SC3, (**C**) SPI5-SC5, (**D**) SPI3-SC7) after TGase crosslinking for various time durations.

As illustrated in Figure 2, the UV-visible absorption spectra of the dual-protein samples underwent notable modifications across different TGase crosslinking durations. Even with TGase crosslinking for 1 h, a significant impact on the UV absorption intensity of proteins was observed when compared to the non-crosslinked samples. Furthermore, a gradual intensification in UV absorption was evident with the extension of the crosslinking duration in all sample groups. This may be due to the conformational changes in SPI or dual-protein samples caused by covalent crosslinking with TGase, exposing and transferring more chromophoric groups to the protein surface [21]. Zhu et al. [9] also reached a similar conclusion in their analysis of TGase crosslinked soy protein using ultraviolet absorption spectroscopy. They found that the TGase crosslinking process exposed certain ultraviolet-absorbing chromophores in soy protein, resulting in an increased intensity of ultraviolet absorption.

### 2.3. Endogenous Fluorescence Spectroscopic Analysis

**Figure 3:**
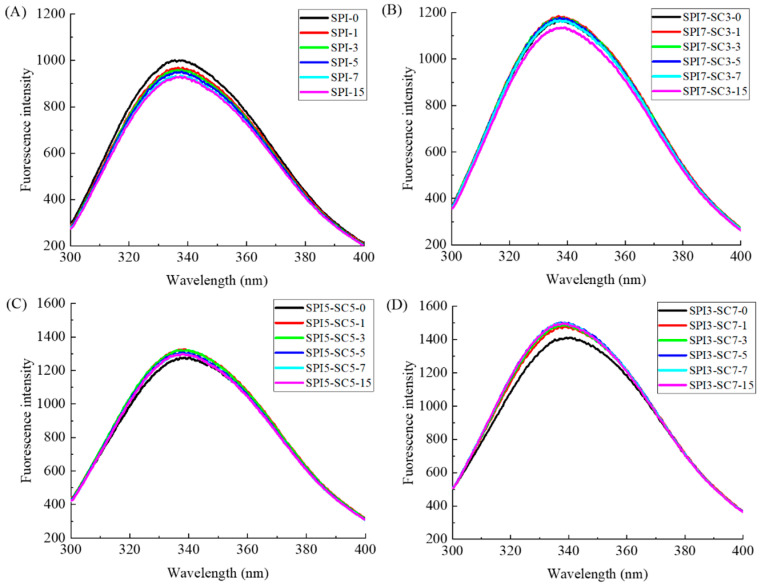
Endogenous fluorescence spectra of dual-protein systems with different SPI-SC proportions ((**A**) SPI, (**B**) SPI7-SC3, (**C**) SPI5-SC5, (**D**) SPI3-SC7) after TGase crosslinking for various time durations.

Endogenous fluorescence spectroscopy is commonly utilized in the study of conformational changes in proteins. The fluorescence peak of both the SPI and dual-protein systems, as shown in Figure 3, occurs at around 340 nm, which aligns with the fluorescence peak of Trp chromophores at approximately 348 nm [22]. This suggested that Trp was the primary chromophore in the SPI or dual-protein systems and that changes in its fluorescence intensity directly reflected variations in Trp residues and the surrounding microenvironment [23].

From Figure 3A, it became apparent that the fluorescence intensity of SPI reduced to varying degrees after different incubation times (1~15 h) with TGase in comparison to the non-crosslinked sample (SPI-0). This decrease was likely attributable to the burial of Trp residues when catalyzed by TGase, leading to a reduction in fluorescence intensity. Additionally, the decreased fluorescence intensity may have resulted from increased electrostatic repulsion, as suggested by Babiker [24]. A similar phenomenon was observed in the SPI7-SC3 sample group (Figure 3B). For the SPI5-SC5 group (Figure 3C), a blueshift in λmax to approximately 338 nm was noted after TGase incubation, accompanied by a higher fluorescence intensity compared to the unlinked SPI5-SC5-0 (λmax = 340.3 nm). Similar trends were observed in the SPI3-SC7 group (Figure 3D) under TGase influence. The combination with casein may contribute to the structural intricacy of the crosslinked proteins, influencing their fluorescence properties. Collectively, these results indicated that TGase crosslinking significantly modified the polar microenvironment around chromophores, such as Trp, affecting their exposure and leading to tertiary structural changes in proteins. These alterations in protein conformation have the potential to induce changes in antigenic epitopes and potentially affect allergenicity.

### 2.4. Surface Hydrophobicity Analysis

Surface hydrophobicity is a key parameter for assessing functional alterations in proteins due to conformational changes. It reflects the dynamic equilibrium between the exposed hydrophobic residues and hydrophilic/hydrophobic groups, serving as an indicative measure of protein unfolding [25]. 

**Figure 4:**
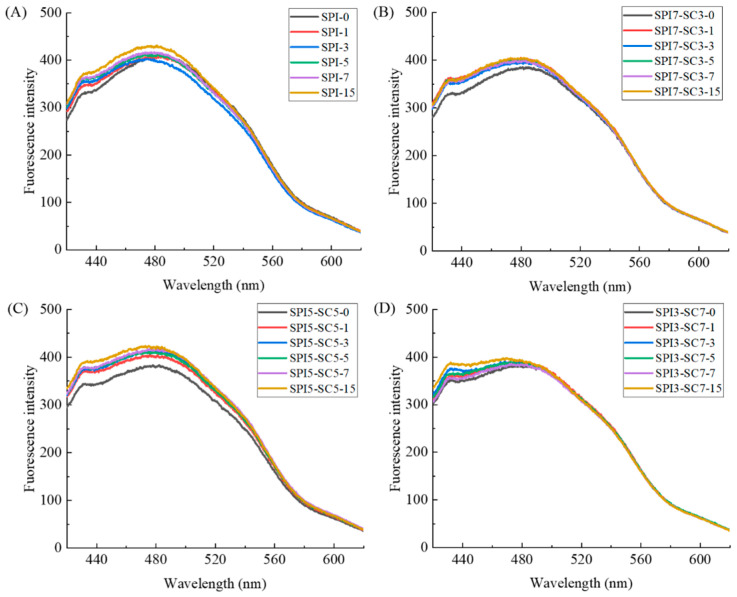
Extrinsic fluorescence spectra of dual-protein systems with different SPI-SC proportions ((**A**) SPI, (**B**) SPI7-SC3, (**C**) SPI5-SC5, (**D**) SPI3-SC7) after TGase crosslinking for various time durations.

As illustrated in Figure 4, progressive crosslinking of TGase resulted in a discernible escalation in exogenous fluorescence intensity for both SPI and dual-protein samples. This observation denoted a simultaneous augmentation in surface hydrophobicity. This might be ascribed to structural modifications induced by TGase catalysis in the SPI or dual-protein samples, leading to the gradual exposure of hydrophobic regions within the molecules and the unfolding of peptide chains [26]. Furthermore, the heightened hydrophobicity may be linked to the deamidation process occurring during TGase crosslinking, resulting in the loss of amino groups. This loss weakens hydrogen bonding between proteins and water, thereby enhancing the exposure of hydrophobic regions and diminishing protein aggregation [9]. Noteworthy is the report by Yang et al. [21], which highlighted a significant increase in the surface hydrophobicity of soy glycinin following TGase crosslinking, closely aligning with the outcomes of the current experiment.

### 2.5. Quantification of Free Sulfhydryl (-SH)

**Figure 5:**
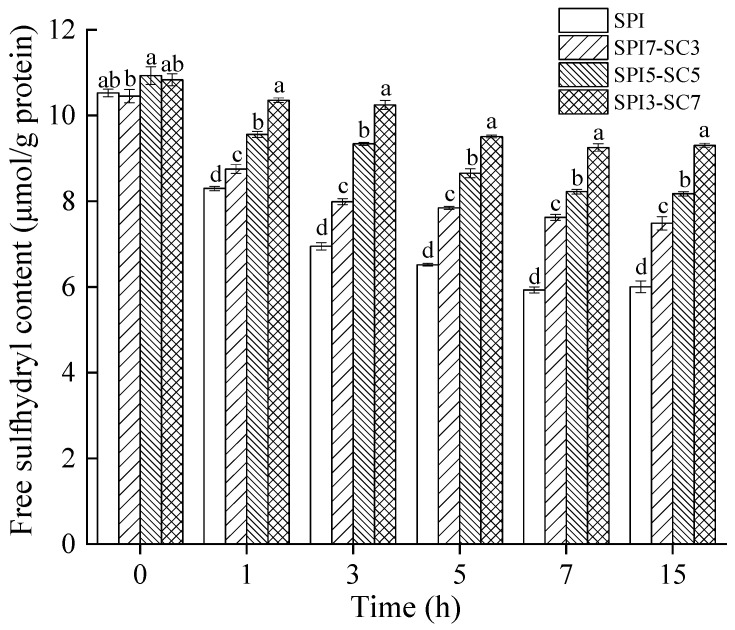
The -SH content of dual-protein systems with different SPI-SC proportions after TGase crosslinking for various time durations. Within the same time point, different lowercase letters indicate a significant difference (*p* < 0.05).

Free sulfhydryl (-SH) groups, primarily located on the protein’s external surface, are crucial for protein functionality. These groups, upon oxidation, can form new disulfide bonds, which are essential for maintaining protein stability [27]. The -SH content after TGase crosslinking at different durations in the dual-protein system is shown in Figure 5. It suggested that there was no significant dissimilarity in -SH content between SPI and dual-protein samples without TGase crosslinking (*p* > 0.05). In contrast, the dual-protein sample with added SC exhibited a significantly higher -SH content compared to the SPI group during the crosslinking process (*p* < 0.05). The -SH content gradually decreased in all sample groups as the duration of TGase crosslinking increased. This could be attributed to TGase facilitating the development of protein polymers through crosslinking, as depicted by the protein electrophoresis findings in Figure 1, causing the surface-exposed -SH groups to be embedded within the polymer [28]. Throughout this process, -SH groups could be converted into -S-S- bonds, leading to a reduction in -SH content. Furthermore, the effect of TGase crosslinking led to the proximity of sulfur-containing amino acids in the newly formed crosslinked peptide chains, fostering disulfide bond formation through oxidation and consequently reducing the-SH content [29]. Previous research by Qin et al. [30] observed a notable decrease in -SH content during the formation of gels with a soy protein isolate and wheat gluten protein mixture when TGase was applied. The reduction in the -SH content was more pronounced under higher high-pressure processing pressures. In a related study, Li et al. [31] reported that TGase addition significantly lowered the -SH content on protein surfaces in soymilk during tofu gel production.

### 2.6. Interactions between Proteins

**Figure 6:**
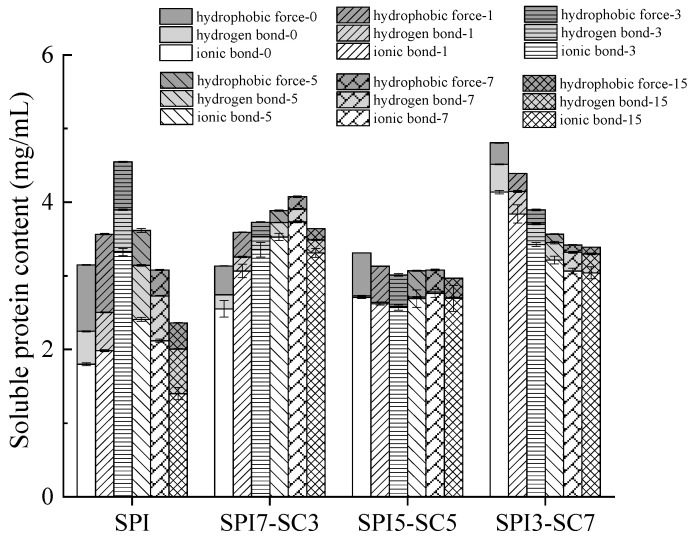
Influence of TGase crosslinking on interaction among proteins (ionic bonds, hydrogen bonds, and hydrophobic forces) in dual-protein systems with different SPI-SC proportions.

The characteristics of proteins are intricately associated with their conformation, a state stabilized by a complex network of intermolecular forces encompassing disulfide bonds, hydrogen bonds, ionic bonds, and hydrophobic interactions [32]. Figure 6 illustrates the presence of hydrophobic interactions, hydrogen bonds, and ionic bonds in the dual-protein systems after TGase crosslinking. The results showed that the unlinked SPI-0 sample exhibited the lowest ionic bond content. A discernible increase in ionic bond content was observed with escalating SC ratios in the dual-protein system, with SPI3-SC7-0 showing the highest value at 4.1 mg/mL. The duration of TGase crosslinking initially led to an increase, followed by a decrease in the ionic bond content in SPI and SPI7-SC3 samples. In contrast, SPI5-SC5 showed negligible changes, while SPI3-SC7 exhibited a gradual decrease in ionic bond content. Hydrogen bonds, though weaker, are critical for stabilizing the protein conformation, particularly in the formation of secondary structures [33]. The hydrogen bond content and hydrophobic forces in SPI peaked after 3 h of TGase crosslinking, while these parameters varied in other groups with prolonged crosslinking durations. This suggested that ionic bonds were predominant in maintaining the structure of dual-protein systems post-TGase crosslinking, complemented by hydrophobic forces and hydrogen bonds.

### 2.7. IgE-Binding Capacity of Soy Proteins in Dual-Protein System

**Figure 7:**
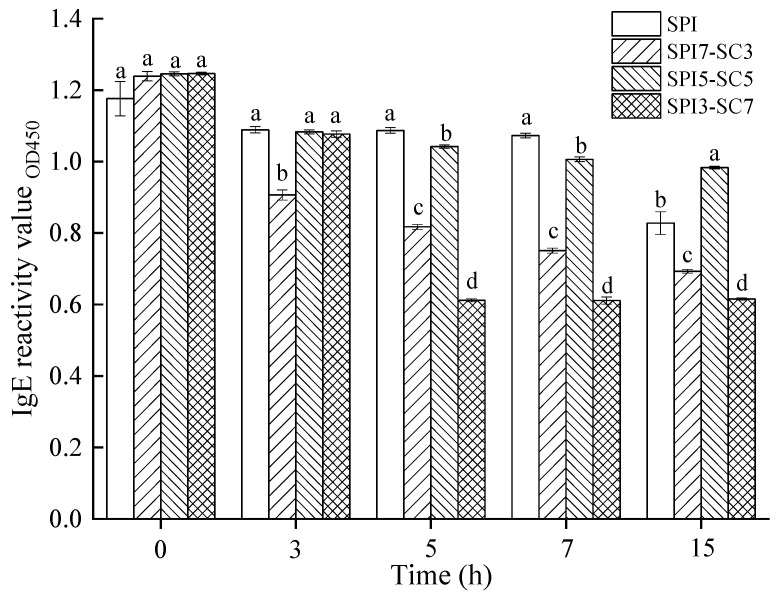
The IgE reactivity of soy protein in the dual-protein systems with different SPI-SC ratios after crosslinking with TGase for various durations. Within the same time point, different lowercase letters indicate a significant difference (*p* < 0.05).

Figure 7 depicts the IgE-binding capacity of soy proteins in dual-protein systems incubated with TGase for varying durations (0, 3, 5, 7, and 15 h). No significant alteration in the IgE-binding capacity of soy proteins was observed in either the dual-protein system or the sole soy protein system (SPI) without TGase crosslinking (*p* > 0.05). However, a progressive decrease in the IgE binding capacity of soy proteins occurred with prolonged TGase crosslinking across all test groups. This trend corresponds with the electrophoretic results presented in Figure 1, implying that extended TGase-mediated catalysis enhances protein crosslinking, potentially masking antigenic epitopes and diminishing antigenicity. At the 3-h mark of TGase crosslinking, the IgE-binding capability of soy proteins in the SPI-3, SPI5-SC5-3, and SPI3-SC7-3 groups did not significantly differ (*p* > 0.05). Nonetheless, these groups displayed notably higher IgE-binding capacity compared to the SPI7-SC3-3 group (*p* < 0.05). Particularly, the IgE-binding ability of the SPI3-SC7 sample decreased significantly, by over 50%, after crosslinking with TGase for 5 h, distinguishing it from the other three groups (*p* < 0.05). Similar patterns were observed in samples crosslinked for 7 and 15 h, with the SPI3-SC7 group demonstrating the lowest IgE-binding capacity, although the decrease was less pronounced. The descending order of IgE-binding capacity in these soy protein samples with extended TGase crosslinking was as follows: SPI > SPI5-SC5 > SPI7-SC3 > SPI3-SC7. This suggested that TGase crosslinking in a dual-protein system, comprising SPI and SC, was more effective in reducing soy protein antigenicity than in a singular protein system (SPI).

The underlying mechanisms involved structural modifications of the dual-protein system, as mentioned above, leading to decreased recognition by IgE antibodies. Additionally, the steric hindrance offered by SC could mask the allergenic sites. This could also be attributed to the formation of soy protein-casein aggregates, supported by the equal dilution of soy protein content across all samples used for ELISA testing. In the dual-protein systems (SPI3-SC7 and SPI7-SC3), antigenic epitopes within the soy protein-casein crosslinks exhibited lower affinity toward soy protein antibodies compared to those within the soy protein crosslinks in the singular soy protein system (SPI). This finding aligns with the research of Li and Damodaran [34]. Xing et al. [19] conducted prior research and reported a significant reduction in the antigenicity of soy protein after 4 h of TGase crosslinking. The reduction was observed to be 67.8% in soymilk and 25.7% in a soymilk and cow milk blend, with statistical significance (*p* < 0.01). Despite a marked decrease in protein bands evident in the electrophoretic profile of Figure 1B after 15 h of TGase treatment (SPI-15), the IgE-binding capacity of soy protein only diminished by approximately 30%. This observation implied that following TGase treatment, the antigenic epitopes of soy protein within the crosslinked polymers of the singular soy protein system (SPI) remained discernible by antibodies. This phenomenon underscores the potential link between protein structure alterations and the state of antigenic epitopes, as discussed by Li, Bu, and Xi [35].

## 3. Materials and Methods

### 3.1. Materials

Soy protein isolate (SPI) and sodium caseinate (SC), each with a minimum protein content of 90% (*w*/*w*), were sourced from Shansong Biological Products Co., Ltd., (Linyi, Shandong, China) and Huaan Biological Products Co., Ltd. (Linxia, Gansu, China), respectively. Transglutaminase (TGase), with enzyme activity quantified at 100 U/g, was procured from Dongsheng Bio-tech Co., Ltd., Taixing, Jiangsu, China. The RIDASCREEN^®^ FAST Soya ELISA test kit (Art. No. R7102) was purchased from R-Biopharm AG (Darmstadt, Germany).

### 3.2. Crosslinked Dual-Protein Samples Preparation

The SPI-SC mixture was prepared in a series of mass ratios, specifically 10:0, 7:3, 5:5, and 3:7, as previously described by Shi et al. [36]. Briefly, a 10 mg/mL protein solution was prepared by dissolving 1 g of SPI in 100 mL of distilled water. This solution, labeled as SPI, constituted an SPI:SC ratio of 10:0. Subsequently, SPI7-SC3 was created by blending 0.7 g of SPI with 0.3 g of SC in 100 mL of distilled water, yielding a SPI:SC ratio of 7:3. Parallel procedures were followed to formulate the SPI5-SC5 and SPI3-SC7 solutions. This ensured that all proteins were adequately dissolved before subsequent processing steps. Each protein solution underwent treatment with TGase at a concentration of 5 Units per gram of protein. This process was conducted at a controlled temperature of 50 °C. Aliquots were systematically collected at 0, 1, 3, 5, 7, and 15 h, corresponding to each ratio of SPI-SC. Following collection, these aliquots were subjected to a deactivation process, which involved heating at 90 °C for 10 min. The resultant samples were classified as SPI-0, SPI-1, SPI-3, SPI-5, SPI-7, SPI-15, and a series of SPI7-SC3 combinations, namely SPI7-SC3-0, SPI7-SC3-1, SPI7-SC3-3, SPI7-SC3-5, SPI7-SC3-7, and SPI7-SC3-15, among others.

### 3.3. Analysis of Protein Composition via Electrophoresis

To analyze changes in protein composition within these dual-protein samples, the SDS-PAGE method was employed. This technique utilizes a 4% (*w*/*v*) stacking gel and a 12% (*w*/*v*) separating gel according to previous literature [37]. Following electrophoresis, gels were subjected to staining and destaining processes and subsequently analyzed through gel image analysis techniques.

### 3.4. Spectrophotometric Analysis of Ultraviolet (UV) Absorption

The UV absorption spectra for each sample, as delineated in Section 3.2, were assessed within the wavelength range from 220 nm to 420 nm, as referenced in the study by Yang et al. [21]. The spectral measurements were replicated thrice to ensure analytical precision.

### 3.5. Analysis of Fluorescence Spectra in Dual-Protein Samples

The fluorescence properties of each dual-protein sample group were analyzed using an F-280 fluorescence spectrophotometer (Gangdong Sci. & Tech. Co., Ltd., Tianjin, China). This analysis involved setting the excitation wavelength at 280 nm and scanning the emission spectrum from 300 nm to 400 nm to acquire fluorescence spectral data, following the method outlined by Liu et al. [38]. To validate the repeatability of these findings, the measurements were conducted in triplicate.

### 3.6. Assessment of Surface Hydrophobicity

The surface hydrophobicity of the dual-protein samples was measured using an 8-Anilino-1-naphthalenesulfonic acid ammonium salt (ANS) fluorescent probe based on the methodology proposed by Yang et al. [39]. Protein concentration for each sample was standardized to 0.5 mg/mL using a 0.1 M phosphate buffer at pH 7.6. An aliquot of 20 µL of ANS (8.0 mmol) was then added to 4 mL of this adjusted protein solution, mixed thoroughly, and incubated at room temperature away from light for a duration of 20 min. The fluorescence intensity was measured across an emission wavelength range from 400 to 650 nm, with an excitation wavelength of 390 nm, at a scanning rate of 240 nm/s, and a slit width set at 5 nm. This process was repeated three times for each sample to ensure consistency.

### 3.7. Determination of Free Sulfhydryl (-SH) Group Content

The free sulfhydryl (-SH) group content in the dual-protein samples was measured following a procedure adapted from Zhang et al. [40]. This assay was replicated three times for each sample.

### 3.8. Elucidation of Intermolecular Interactions

To investigate the nature of intermolecular interactions within the proteins, including hydrogen bonds, ionic bonds, and hydrophobic forces, the freeze-dried dual-protein samples were dissolved in three distinct solutions following the protocol by Shi et al. [36]. Multiple extraction steps were carried out under the same conditions: dissolution (3000 rpm, 2 min), extraction (1 h at 4 °C), and centrifugation (10,000× *g*, 20 min). These steps were referred to as “extraction” in the subsequent description. 

Five milligrams of freeze-dried sample were mixed with 1 mL of solution A (0.6 mol/L NaCl), followed by sequential extractions with solutions B (0.6 mol/L NaCl with 1.5 mol/L urea) and C (0.6 mol/L NaCl with 8 mol/L urea). Supernatants S1, S2, and S3 were collected, and their protein contents were quantified using the BCA protein concentration assay kit, indicating the presence of ionic bonds, hydrogen bonds, and hydrophobic forces, respectively. All assays were conducted in triplicate.

### 3.9. Immunoassay for Assessing IgE Reactivity

The IgE reactivity of soy protein in the TGase crosslinked dual-protein samples was evaluated using the RIDASCREEN^®^ FAST Soya ELISA Sandwich Immunoassay Kit, according to the methodologies established in previous research [34]. The antibodies in the kit are specific to soy and exhibit no cross-reactivity with animal-based proteins (casein, β-lactoglobulin, egg white proteins, etc.). The preparation and analysis of the samples followed the procedural guidelines provided with the kit. Soy protein concentrations in each sample group were adjusted to 20 μg/mL using a sample dilution solution to align with the kit’s linear detection range.

A volume of 100 µL of the diluted specimen was pipetted into microtiter plate wells that had been previously coated with the primary antibody. This was followed by an incubation period of 10 min at a controlled temperature of 25 °C. To ensure the removal of non-specifically bound proteins, the wells underwent a double-washing procedure using a suitable wash buffer. Thereafter, each well received 100 µL of a horseradish peroxidase-conjugated secondary antibody, with a subsequent incubation for 10 min at 25 °C. Following incubation, the wells were subjected to further washing before the introduction of 100 µL of the substrate solution. This reaction proceeded for 10 min at 25 °C in an environment shielded from light, after which it was terminated by the addition of 100 µL of stop solution. The absorbance was then quantified at 450 nm by a microplate reader (ReadMax 1900, Shanghai Shanpu Biotechnology Co. LTD., Shanghai, China). The obtained absorbance values demonstrated a direct correlation with the concentration of soy protein antigen in the samples. Each sample was assayed in duplicate to ensure the reliability of the measurements.

### 3.10. Statistical Analysis

Statistical analysis was performed using SPSS software 16.0. The data were subjected to one-way analysis of variance (ANOVA), followed by Duncan’s multiple range test to identify significant differences among mean values (*p* < 0.05). Graphical data representations were created using Origin 9.0.

## 4. Conclusions

The results of this study revealed that the dual-protein systems (SPI7-SC3 and SPI3-SC7) conferred a notable advantage in mitigating the antigenicity of soy protein following TGase crosslinking. The decrease in the IgE-binding capacity of soy protein across all evaluated dual-protein samples was attributed to alterations in the overall dual-protein system rather than changes in SPI alone. TGase crosslinking resulted in the formation of high-molecular-weight soy protein-casein aggregates, leading to significant conformational alterations within the dual-protein matrix. These alterations include increased exposure to internal ultraviolet absorbance groups, heightened surface hydrophobicity, and reduced free sulfhydryl content. These findings implied that TGase-mediated crosslinking induced changes in the conformation of allergenic proteins within the soy cow milk dual-protein system, potentially affecting their antigenic epitopes and allergenic properties. This study highlights the potential of using TGase crosslinking in dual-protein systems to produce hypoallergenic food products, especially for individuals with soy protein allergies. The combination of SPI and SC in varying ratios demonstrates the efficacy of this approach in mitigating allergenic responses. Future studies could extend this research by including similar analyses for casein to provide a more comprehensive understanding of the dual-protein system’s impact on both proteins’ allergenicity. Additionally, future research should explore the in vivo implications of these findings and further investigate the interactions between different protein components in mixed-protein matrices to enhance our understanding of allergenicity reduction mechanisms. 

## Data Availability

The data presented in this study are available on request from the corresponding author due to reasonable request.
